# Podocalyxin as a Prognostic Marker in Gastric Cancer

**DOI:** 10.1371/journal.pone.0145079

**Published:** 2015-12-16

**Authors:** Alli Laitinen, Camilla Böckelman, Jaana Hagström, Arto Kokkola, Christian Fermér, Olle Nilsson, Caj Haglund

**Affiliations:** 1 Department of Surgery, University of Helsinki and Helsinki University Hospital, Helsinki, Finland; 2 Research Programs Unit, Translational Cancer Biology, University of Helsinki, Helsinki, Finland; 3 Department of Pathology and Oral Pathology, University of Helsinki and Helsinki University Hospital, Helsinki, Finland; 4 Fujirebio Diagnostics AB, Gothenburg, Sweden; 5 Onson Consulting, Gothenburg, Sweden; INRS, CANADA

## Abstract

**Background:**

Podocalyxin-like 1 (PODXL) is a cell-adhesion glycoprotein associated with aggressive tumor phenotype and poor prognosis in several forms of cancer. The aim of this study was to investigate PODXL expression in gastric cancer by use of two different antibodies.

**Methods:**

By tumor-tissue microarrays and immunohistochemistry we evaluated PODXL expression in tumor specimens from 337 patients who underwent surgery for gastric adenocarcinoma at Helsinki University Hospital. We used two different antibodies: HPA2110, which is a polyclonal antibody and an in-house monoclonal antibody called HES9, to investigate the association of PODXL expression with clinicopathologic variables and patient survival.

**Results:**

PODXL staining was positive by the polyclonal antibody in 153 (57.5%) cases and by the monoclonal antibody in 212 (76%). Polyclonal antibody expression was associated with intestinal cancer type (p<0.001). Monoclonal antibody staining was associated with age over 66 (p = 0.001), with intestinal cancer (p<0.001), and with small tumor size (≤ 5 cm; p = 0.024). Both antibodies were associated with high S-phase fraction (p = 0.022; p = 0.010), and high tumor proliferation index (Ki-67; p = 0.003; p = 0.001). PODXL positivity by the polyclonal antibody indicated reduced gastric-cancer-specific 5-year survival of 24.0% (95% CI 16.9–31.1), compared to 43.3% (95% CI 33.7–52.9) for patients with PODXL negativity (p = 0.001). The result remained significant in multivariable analysis (HR = 3.17; 95% CI 1.37–7.34, p = 0.007).

**Conclusion:**

In gastric cancer, PODXL expression by the polyclonal antibody HPA2110 is an independent marker of poor prognosis.

## Introduction

In the last decades, the incidence of gastric cancer has declined, especially in Western countries, apparently due to the declining incidence of *Helicobacter pylori*, to better hygiene, and to less crowded living. Because gastric cancer has a poor prognosis, it is still globally the second most common cause of cancer-related death [1]. The 5-year survival rate, despite curative surgery, is only about 10–30% [2]. The high mortality is mostly due to late diagnosis.

The UICC Tumor Node Metastasis (TNM) classification is by far the most consistent prognostic classification system today. However, even within the same stage group tumors, the course of disease can vary. Creating methods for more accurate assessment of the neoplasia aggressiveness would be of value when evaluating the prognosis of individual patients with gastric cancer.

Podocalyxin-like 1 (PODXL) is an anti-adhesive transmembrane glycoprotein belonging to the CD34 family. It is expressed normally by kidney podocytes [3], haemopoietic progenitor cells [4], vascular endothelia [5], and breast epithelial cells [6].

The clinical significance of PODXL in cancer progression has been investigated in various carcinoma types, first as a stem cell marker in testicular cancer [7]. A later finding is that tumor-cell-specific PODXL expression is associated with a more aggressive phenotype and adverse outcome in several cancer types, for example, in breast [6], prostate [8], ovarian [9], colorectal [10–12], urothelial bladder [13], pancreatic [14], and periampullary cancer [15].

The aim of this study was to investigate PODXL expression in gastric cancer to reveal its possible role in aggressiveness and prognosis. We decided to use two PODXL antibodies recognising different epitopes: a monoclonal in-house HES9 antibody and a commercially available polyclonal HPA2110 antibody. Recently we learned that these two antibodies are independent markers of poor prognosis in colorectal cancer [12]. The two antibodies can recognise two groups of colorectal cancer patients, both of whom have a poor prognosis; combined use of these antibodies revealed a patient group with even worse prognosis [16].

## Materials and Methods

### Patients

The study comprised 337 consecutive patients who underwent surgery for histologically verified gastric adenocarcinoma at the Department of Surgery, Helsinki University Hospital, from 1983 to 1999. Diagnosis and staging according to the UICC classification provided 100 (29.7%) stage IA-IB, 41 (12.2%) stage II, 96 (28.5%) stage IIIA-IIIB, and 100 (29.7%) stage IV patients. Lymph-node metastases occurred in 184 (55%) and distant metastases in 93 (28%) cases. Median age was 66 years (range 30–87), and 163 (48%) were women and 174 (52%) men. Total or partial gastrectomy with extended (D2-D2+) lymphadenectomy was performed in 34 (10%) patients, total gastrectomy with D1-lymphadenectomy in 161 (48%), and subtotal gastrectomy with D1-lymphadenectomy in 142 (42%). In total, 143 (43%) patients were operated on with curative intent, whereas 176 (52%) underwent palliative surgery. None received neoadjuvant treatment, but 32 (9%) patients received postoperative adjuvant treatment (28 chemotherapy, 2 radiotherapy; 2 received both). Survival data and cause of death until November 2013 came from patient records, the Population Register Centre of Finland, and Statistics Finland.

The study was approved by the Surgical Ethics Committee of Helsinki University Hospital (Dnro HUS 226/E6/ 06, extension TMK02 §66 17.4.2013) and the National Supervisory Authority of Welfare and Health gave permission to use the tissue samples without individual consent in this retrospective study (Valvira Dnro 10041/06.01.03.01/2012).

### Tissue samples

Formalin-fixed and paraffin-embedded surgical tissue samples came from the archives of the Department of Pathology. The patient tissues were de-identified and analyzed anonymously. Histopathologically representative areas of tumor specimens were defined and marked on haematoxylin- and eosin-stained slides. Three cores from each tumor block were sampled with 0.6-mm punchers and embedded in a new paraffin block by a semi-automatic tissue microarrayer (Tissue Arrayer 1, Beecher Instruments Inc., Silver Spring, MD, USA). Of the six tissue-array blocks prepared, each contained 24–192 tumor samples. Sections of 4 μm each were cut and processed for immunohistochemistry.

### Antibodies

We compared two PODXL antibodies against different epitopes. The polyclonal antibody (HPA2110, Atlas Antibodies, Stockholm, Sweden) was raised against a peptide with the amino acid residues 278–415 of PODXL, and the monoclonal in-house antibody HES9 [12] recognises the amino acid residues 189–192 of PODXL. Both epitopes occur in the extracellular part of the PODXL molecule. The epitope sequence of the HPA2110 matches three protein coding PODXL splice variants with 100% (PODXL 001, 005, and 201, The Human Protein Atlas). The fourth splice variant matches with 87% (PODXL 202). The epitope sequences of the HES9 matches 100% with all splice variants. The antibodies are described in detail [12,17,18].

### Immunohistochemistry

Sections were fixed on slides and dried for 12 to 24 hours at 37°C, then were deparaffinized in xylene and rehydrated through gradually decreasing concentrations of ethanol to distilled water. For antigen retrieval, sections were treated in a PreTreatment Module (Lab Vision Corp., Fremont, CA, USA) in Tris-HCl buffer (pH 8.5) for 20 minutes at 98°C. Sections were stained in an Autostainer 480 (Lab Vision) by the Dako REAL EnVision Detection System, Peroxidase/DAB+, Rabbit/Mouse (Dako, Glostrup, Denmark). The slides were treated for 5 minutes with 0.3% Dako REAL Peroxidase-Blocking Solution to block endogenous peroxidases. Subsequently, slides were incubated for 1 hour with a polyclonal (dilution 1:250 = 2.5 μg/ml) or monoclonal antibody (dilution 1:500 = 5 μg/ml) at room temperature. As a positive control for each staining series we used samples from renal tissue.

### Scoring of immunoreactivity

HPA2110 and HES9 immunopositivity was graded in all available tumor cores. Cytoplasmic PODXL and HES9 immunopositivity was scored as 0 to 3 based on intensity of cancer cell immunoreactivity, and the highest score of the three cores served for further analysis. Negative immunoreactivity was scored as 0, diffuse weak cytoplasmic positivity as 1, moderately positive or focally strongly positive intensity as 2, and homogeneously strong intensity as 3. All samples were scored independently by two researchers (A.L. and J.H.) blinded to clinical status and outcome data. Specimens with discordant scores were re-evaluated, and the consensus score served for further analysis. We were able to process and score HPA2110 staining in 266 and HES9 staining in 279 samples.

### Statistical analysis

Associations between HPA2110 and HES9 positivity and clinicopathologic variables were assessed by the chi-square test or Fisher’s exact test. Correlations between the two podocalyxin antibodies were assessed by the Spearman correlation test. Disease-specific survival was calculated from date of surgery to death from gastric cancer.

Survival curves were constructed according to the Kaplan-Meier method and compared with the logrank test. For univariable and multivariable survival analysis, the Cox proportional hazard model had the following covariates entered: age, gender, stage, grade, Lauréns classification, tumor size, tumor location, PODXL expression determined by HPA2110 staining and HES9 staining, DNA ploidy, S-phase fraction, Ki-67 expression, and p53 expression. Stage, grade, Lauréns classification, tumor size, DNA ploidy, S-phase fraction, and expression of PODXL, HES9, Ki-67, and p53 were entered as categorical covariates. DNA ploidy and S-phase fraction had been assessed earlier by flow cytometry, and p53, and Ki-67 tissue expression by immunohistochemistry [19,20]. A p-value of <0.05 was considered statistically significant. All statistical analyses were done with IBM SPSS Statistics version 20.0 for Mac (IBM Corporation, Armonk, NY, USA).

## Results

### Immunohistochemistry

**Fig 1:**
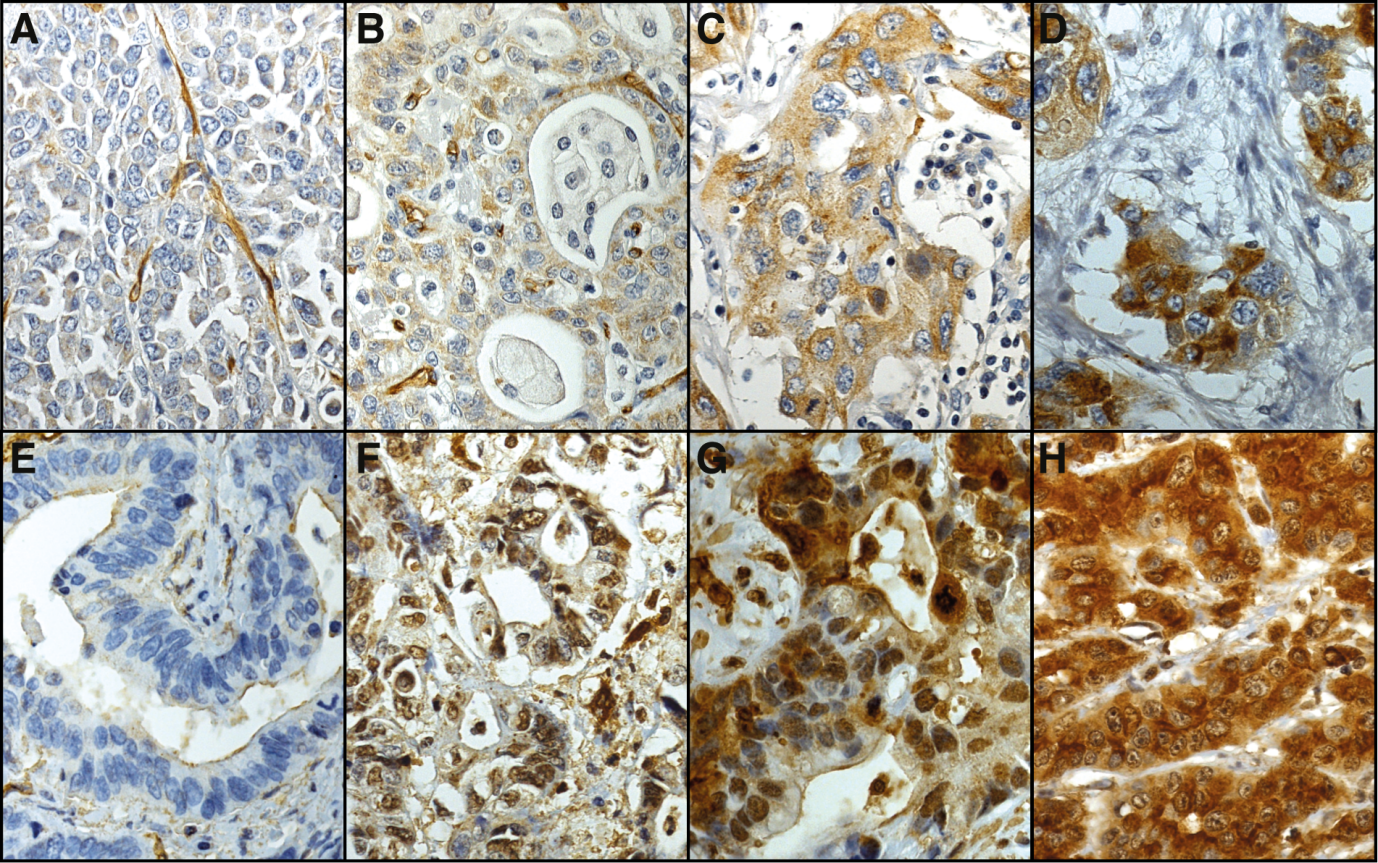
Representative images of HPA2110 and HES9 staining representing gastric cancer tumors with negative (A, E), weak (B, F), moderate (C, G), and strong (D, H) staining in a varying proportion of tumor cells. Images A-D stained with the polyclonal HPA2110 antibody and E-H with the monoclonal HES9 antibody. Original magnification 40x.

Cytoplasmic HPA2110 reactivity, evaluated in 266 cases, was negative in 113 (42.5%), weakly positive in 120 (45.1%), moderately positive in 29 (10.9%), and strongly positive in 4 (1.5%) cases. Of the 279 cases successfully stained for cytoplasmic expression by HES9, 67 (24.0%) were negative, 137 (49.1%) weakly positive, 54 (19.4%) moderately positive, and 21 (7.5%) strongly positive. In the final analysis, weak to strongly positive HPA2110 and HES9 immunoreactivity (scores 1–3) was regarded as positive expression. Representative images of immunostainings are in Fig 1. Both HPA2110 and HES9 stained evenly throughout the cytoplasm, with nuclear nor cell membranous immunopositivity noticeable. The cytoplasmic expression of podocalyxin by the two different antibodies HPA2110 and HES9 correlated (r_s_ = 0.455, p<0,001, Spearman's rank correlation test).

### Association of HPA2110 and HES-9 with clinicopathological variables and biomarkers

The associations of HPA2110 and HES9 with clinicopathological variables and molecular biomarkers were as follows: positive (scores 1–3) HPA2110 staining was associated with intestinal cancer type (p<0.001), positive HES9 staining was associated with age over 66 (p = 0.001), with intestinal cancer (p<0.001), and with small (≤ 5 cm) tumor size (p = 0.024). Both HPA2110 and HES9 were associated with high S-phase fraction (SPF, p = 0.022; p = 0.010) and high proliferation index of the tumor (Ki-67, p = 0.003; p = 0.001) (Tables 1 and 2).

**Table 1:** Association of HPA2110 and HES9 with clinicopathological variables in gastric cancer patients.

	HPA2110						HES9					
		Negative		Positive				Negative		Positive		
Clinicopathological												
variable	n	n	%	n	%	p-value	n	n	%	n	%	p-value
Age, years												
< 66	123	58	47.2	65	52.8	0.153	126	42	33.3	84	66.7	0.001
≥ 66	143	55	38.5	88	61.5		153	25	16.3	128	83.7	
Gender												
Male	137	52	38.0	85	62.0	0.124	143	30	21.0	113	79.0	0.224
Female	129	61	47.3	68	52.7		136	37	27.2	99	72.8	
Stage												
IA-IB	62	30	48.4	32	51.6	0.709	67	17	25.4	50	74.6	0.594
II	35	13	37.1	22	62.9		36	8	22.2	28	77.8	
IIIA-IIIB	83	34	41.0	49	59.0		87	17	19.5	70	80.5	
IV	86	36	41.9	50	58.1		89	25	28.1	64	71.9	
pT-classification												
pT1	38	21	55.3	17	44.7	0.250	39	8	20.5	31	79.5	0.684
pT2	39	17	43.6	22	56.4		43	13	30.2	30	69.8	
pT3	136	51	37.5	85	62.5		143	32	22.4	111	77.6	
pT4	53	24	45.3	29	54.7		54	14	25.9	40	74.1	
pN0	107	48	44.9	59	55.1	0.465	112	28	25.0	84	75.0	0.812
pN1	80	36	45.0	44	55.0		89	19	21.3	70	78.7	
pN2	79	29	36.7	50	63.3		77	19	24.7	58	75.3	
pM0	185	80	43.2	105	56.8	0.704	195	44	22.6	151	77.4	0.388
pM1	81	33	40.7	48	59.3		84	23	27.4	61	72.6	
Laurén classification												
Intestinal	120	37	30.8	83	69.2	<0.001	120	16	13.3	104	86.7	<0.001
Diffuse	146	76	52.1	70	47.9		159	51	32.1	108	67.9	
Grade												
1	17	5	29.4	12	70.6	0.579	16	3	18.8	13	81.3	0.454
2	39	11	28.2	28	71.8		40	4	10.0	36	90.0	
3	50	17	34.0	33	66.0		53	11	20.8	42	79.2	
4	7	4	57.1	3	42.9		7	0	0.0	7	100.0	
5	1	0	0.0	1	100.0		1	0	0.0	1	100.0	
Tumor size, cm												
≤ 5	140	56	40.0	84	60	0.335	145	26	17.9	119	82.1	0.024
> 5	122	56	45.9	66	54.1		129	38	29.5	91	70.5	

Abbreviations: HPA2110 = polyclonal antibody, HES9 = monoclonal antibody

**Table 2:** Association of HPA2110 and HES9 with molecular biomarkers in gastric cancer patients.

	HPA2110						HES9					
		Negative		Positive				Negative		Positive		
Biomarker												
	n	n	%	n	%	p-value	n	n	%	n	%	p-value
SPF 7.6												
< 7.6	99	49	49.5	50	50.5	0.022	109	35	32.1	74	67.9	0.010
≥ 7.6	120	41	34.2	79	65.8		125	22	17.6	103	82.4	
DNA ploidy												
Diploid	171	79	46.2	92	53.8	0.084	186	51	27.4	135	72.6	0.080
Aneuploid	73	25	34.2	48	65.8		71	12	16.9	59	83.1	
p53												
≤ 20%	178	75	42.1	103	57.9	0.871	186	47	25.3	139	74.7	0.488
> 20%	88	38	43.2	50	56.8		93	20	21.5	73	78.5	
Ki-67												
< 10%	69	38	55.1	31	44.9	0.003	76	27	35.5	49	64.5	0.001
≥ 10%	155	53	34.2	102	65.8		161	27	16.8	134	83.2	

Abbrevations: HPA2110 = polyclonal antibody, HES9 = monoclonal antibody; SPF = S-phase fraction

### Survival analyses

**Fig 2:**
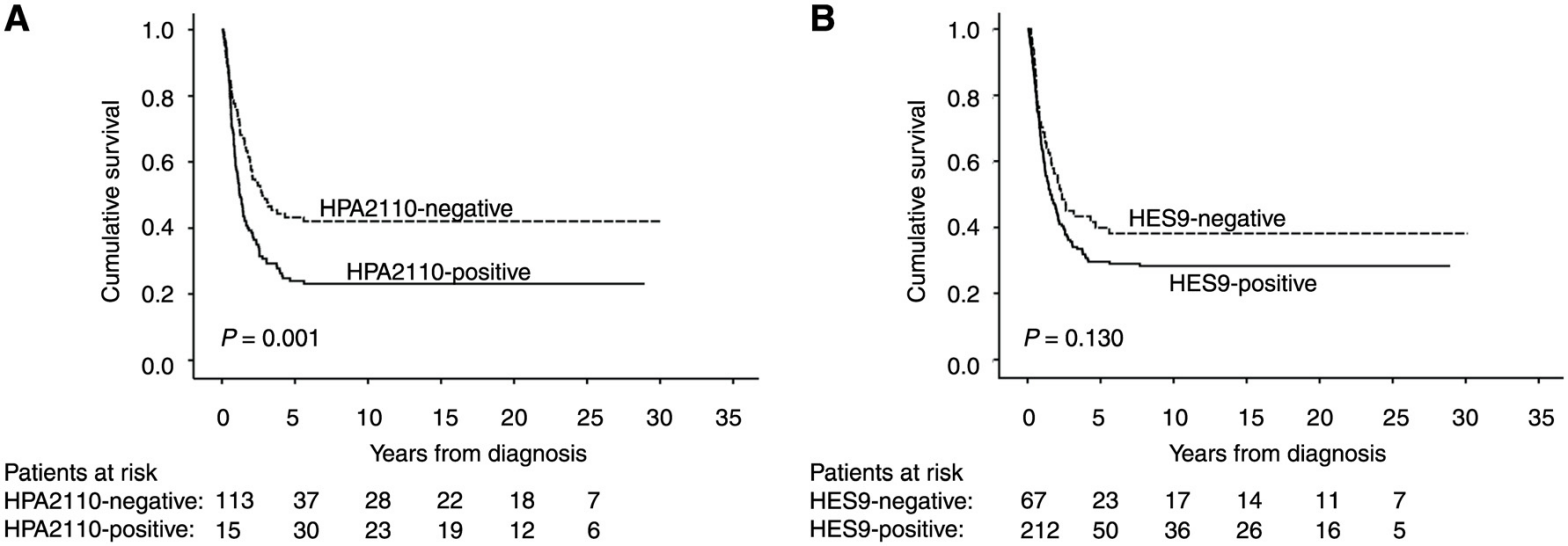
Gastric-cancer-specific survival according to the Kaplan–Meier method. (A) HPA2110 and (B) HES9 expression and survival in gastric cancer patients.

Positive HPA2110 expression indicated a gastric cancer-specific 5-year survival of 24% (95% CI 16.9–31.1), compared to patients with negative HPA2110 staining with 5-year survival of 43% (95% CI 33.7–52.9) (p = 0.001 log-rank test) (Fig 2 and Table 3). The gastric cancer-specific 5-year survival of patients with HES9-positive tumor staining was 30% (95% CI 23.1–36.1), and for those with negative staining, 40% (95% CI 27.7–52.1; p = 0.130 log-rank test) (Fig 2).

**Table 3:** Kaplan-Meier analysis for disease-specific survival stratified for subgroups of gastric cancer patients.

	5-year cumulative survival (95% CI)			
Subgroup	All patients	HPA2110-negative	HPA2110-positive	p-value
HPA2110	36.3 (31.0–41.6)	43.3 (33.7–52.9)	24.0 (16.9–31.1)	0.001
Age, years				
< 66	44.7 (36.9–52.5)	53.9 (40.8–67.0)	27.8 (16.8–38.8)	0.006
≥66	27.5 (20.2–34.8)	30.2 (16.7–43.7)	20.7 (11.5–29.9)	0.103
Gender				
Male	33.2 (25.8–40.6)	43.9 (29.2–58.6)	16.9 (8.4–25.3)	0.002
Female	39.5 (31.7–47.3)	42.6 (29.7–55.5)	32.9 (21.3–44.5)	0.170
Lauréns classification				
Intestinal	35.9 (27.5–44.3)	43.5 (26.4–60.6)	27.0 (16.8–37.2)	0.150
Diffuse	36.7 (29.6–43.8)	43.1 (31.3–54.9)	20.6 (10.8–30.4)	0.001
Stage				
IA-B	86.1 (79.0–93.2)	96.7 (90.2–1.03)	78.8 (63.7–93.9)	0.048
II	40.0 (24.3–55.7)	41.7 (13.9–69.5)	32.9 (12.5–53.3)	0.526
IIIA-B	13.5 (6.2–20.8)	30.5 (12.5–53.3)	6.5 (0–13.6)	0.005
IV	3.6 (0–7.5)	6.4 (0–15.0)	2.2 (0–6.5)	0.057
pT-classification				
pT1	87.4 (78.6–96.2)	95.2 (86.2–1.04)	80.2 (60.0–1.00)	0.185
pT2	61.5 (48.7–74.2)	54.1 (28.4–79.8)	55.5 (33.7–77.3)	0.960
pT3	19.4 (12.7–26.1)	31.7 (17.8–45.6)	13.9 (6.0–21.7)	0.010
pT4	2.2 (0–6.1)	8.7 (0–20.3)	0 (0–0)	0.152
pN-classification				
pN0	67.5 (59.7–75.3)	77.6 (65.3–89.9)	54.5 (41.2–67.8)	0.022
pN+	9.9 (5.4–14.4)	17.9 (7.9–27.9)	4.6 (0.2–8.9)	0.002
Tumor size, diameter				
< 5 cm	55.5 (47.9–63.1)	69.2 (56.7–81.7)	36.6 (25.6–47.6)	0.001
≥ 5 cm	12.5 (6.8–18.2)	17.8 (7.0–28.6)	8.7 (1.6–15.8)	0.006
Ploidity				
Diploid	40.1 (33.4–46.8)	48.3 (36.7–59.9)	24.8 (15.6–34.0)	0.001
Aneuploid	14.2 (6.4–22.0)	17.1 (8.3–33.4)	13.6 (3.4–23.8)	0.735
SPF				
< 7.6	47.6 (39.0–56.2)	54.2 (39.3–69.1)	33.0 (19.5–46.5)	0.036
≥ 7.6	19.9 (13.0–26.8)	31.7 (17.0–46.5)	12.9 (5.3–20.59	0.012
Ki-67				
≤ 10%	32.3 (22.1–42.5)	39.6 (23.5–55.7)	16.1 (2.4–29.8)	0.060
> 10%	29.8 (22.5–37.1)	36.7 (23.0–50.4)	22.1 (13.7–30.5)	0.043
p53				
≤ 20%	41.5 (34.8–48.2)	49.8 (37.8–61.8)	28.4 (19.4–37.4)	0.004
> 20%	24.7 (15.9–33.5)	29.4 (13.9–44.9)	14.0 (3.6–24.4)	0.071

Abbreviations: HPA2110 = polyclonal antibody, CI = confidence interval; SPF = S-phase fraction

**Fig 3:**
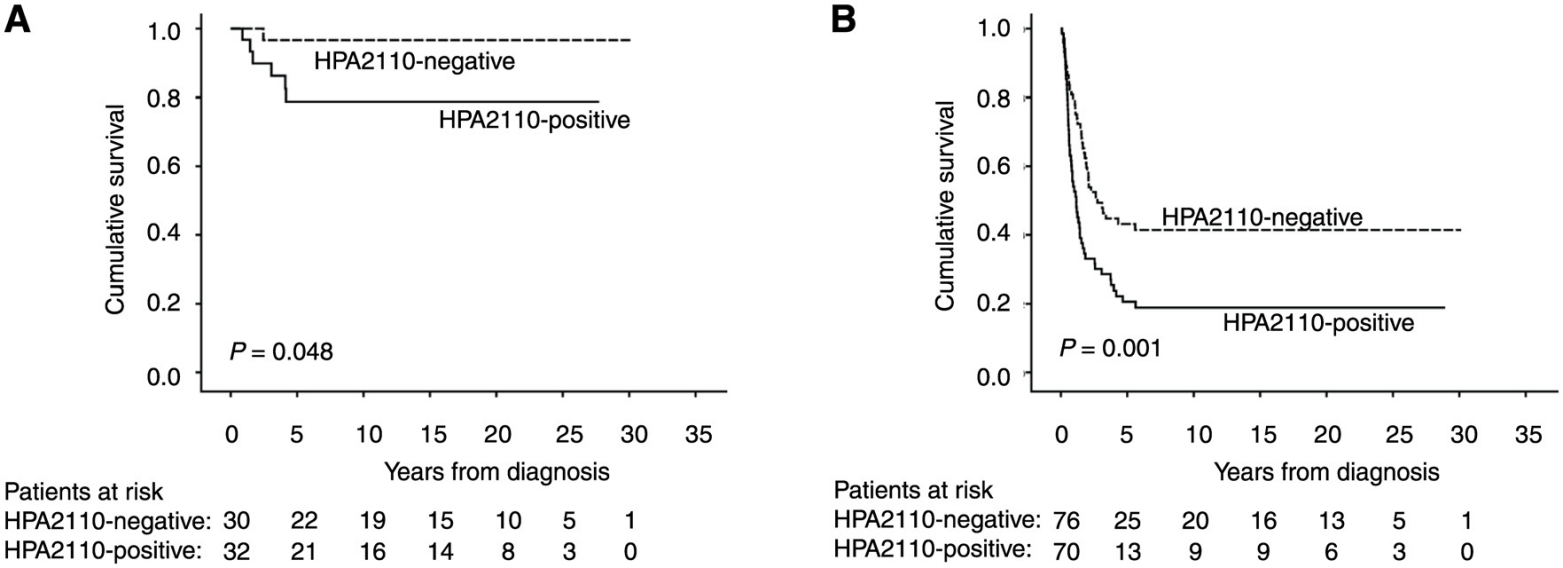
The expression of HPA2110 in the subgroups of (A) stage I patients, and (B) diffuse-type in gastric cancer.

In subgroup analysis, HPA2110 was a significant marker of poor prognosis in groups of younger patients (age under 66 years) (p = 0.006), and for male gender (p = 0.002), diffuse-type cancer (p = 0.001), and stage I patients (p = 0.048) (Fig 3 and Table 3). In the other subgroups studied, HPA2110 did not serve as a prognostic marker.

The HPA2110 expression remained significant in multivariable analysis (HR = 3.17; 95% CI 1.37–7.34; p = 0.007; Table 4). Other independent prognostic factors in multivariable analysis were stage, grade, and DNA ploidy.

**Table 4:** Cox regression analysis for disease-specific survival of gastric cancer patients.

	Univariable survival analysis			Multivariable survival analysis		
Variable	Hazard ratio	95% CI	p-value	Hazard ratio	95% CI	p-value
Age, years						
< 66	1.00					
≥ 66	1.55	1.17–2.04	0.002			
Gender						
Male	1.00					
Female	0.86	0.66–1.14	0.302			
Stage						
I	1.00					
II	5.50	2.87–10.6	<0.001	1.35	0.22–8.44	0.75
III	11.6	6.60–20.3	<0.001	9.55	2.07–44.0	0.004
IV	27.5	15.6–48.5	<0.001	32.2	6.21–167	<0.001
Grade						
1	1.00					
2	1.14	0.59–2.22	0.694			
3	1.55	0.84–2.85	0.161			
4	2.51	1.00–6.33	0.051			
Lauréns classification						
Intestinal	1.00					
Diffuse	1.02	0.77–1.35	0.893			
Tumor size						
≤ 5 cm	1.00					
> 5 cm	3.44	2.57–4.60	<0.001			
Tumor location						
Upper third	1.00					
Middle third	0.55	0.37–0.81	0.002			
Lower third	0.74	0.51–1.06	0.102			
Diffuse	2.01	1.18–3.44	0.010			
Stump	1.26	0.46–3.50	0.655			
HPA2110						
Negative	1.00					
Positive	1.69	1.23–2.32	0.001	3.17	1.37–7.34	0.007
HES9						
Negative	1.00					
Positive	1.32	0.92–1.88	0.131			

Abbreviations: HPA2110 = polyclonal antibody, HES9 = monoclonal antibody, CI = confidence interval

## Discussion

We show here that cancer-specific 5-year survival was significantly better for gastric cancer patients having a PODXL-negative tumor. Actually, among stage 1 patients with PODXL-negative tumors, only one patient died from cancer. In multivariable analysis, positive PODXL expression served as an independent marker of poor prognosis. Positive PODXL expression both by the polyclonal antibody HPA2110 and the monoclonal antibody HES9 was associated with high SPF by flow cytometry and Ki-67 staining, both being markers of high proliferation of cancer cells and thus of worse prognosis.

Positive expression of PODXL by both antibodies was associated with intestinal cancer type, but a PODXL-negative tumor proved not to be a prognostic marker in intestinal cancer. Conversely, cancer-specific 5-year survival in the subgroup of diffuse type cancer was significantly worse for patients with a PODXL-positive tumor. Generally, diffuse type cancer has been associated with worse prognosis than has the intestinal type [21].

To the best of our knowledge, we are the first to report a prognostic value for PODXL in gastric cancer. This has been evident for several other cancer types, like breast [6], prostate [8], ovarian [9], colorectal [10–12], urothelial bladder [13], pancreatic [14], and periampullary cancer [15]. A study on colorectal cancer show concordance between primary tumors and corresponding lymph node metastases in individual patients, and PODXL expression also remains unaffected by neoadjuvant radiation therapy. These findings support the clinical utility of PODXL as a marker of poor prognosis [22]. Here we did not study PODXL expression in lymph node metastases in gastric cancer, and none of the patients in this cohort received preoperative chemotherapy, although it is currently rather routine. These effects of preoperative chemotherapy on PODXL expression in gastric cancer needs further evaluation.

We used two antibodies recognizing PODXL, a commercially available polyclonal antibody HPA2110 and an in-house monoclonal antibody HES9 [16]. Immunoexpression by the monoclonal antibody HES9 was stronger than by the polyclonal antibody. Both antibodies stained evenly throughout the cytoplasm, and neither stained nuclei nor cell membrane. The variety in staining intensity and distribution was slighter when using the polyclonal antibody, making the scoring more difficult. Basically PODXL is a transmembrane molecule, and therefore it was surprising not to find it on cell membranes in gastric cancer cells by either antibody. The meaning of cytoplasmic, non-membraneous, expression of PODXL, its role and function, need further evaluation.

These different antibodies we used recognize unequal parts of the PODXL molecule, probably explaining why their expression in some of the specimens differed and results were not identical. Kaprio et al. [16], used these same antibodies in colorectal cancer, and found an interesting difference between the staining patterns. The polyclonal antibody positivity was membranous [9,10] but, the monoclonal antibody positivity was evident mainly in the cytoplasm. In colorectal cancer, strong positivity with both the monoclonal and the polyclonal antibody revealed a subgroup of patients with even worse survival. We did not find this kind of correlation in gastric cancer samples. Overall, positivity among gastric cancer patients was lower than in colon cancer patients. In colorectal cancer about 94% of the cases were positive for PODXL with these two antibodies, whereas in gastric cancer positivity appeared in 76% cases with the monoclonal antibody and in 58% with the polyclonal antibody. The frequency of PODXL positivity in gastric cancer was close to that seen in breast and ovarian cancer (40% and 67%) [6,9].

PODXL is a transmembrane glycoprotein. It has been shown that mucins, that are also glycoproteins, play a role in epithelial to mesenchymal transition (EMT) [23,24]. The role of PODXL in cancer is unknown but it can be speculated that also PODXL may have a role in EMT. EMT may explain why patients with high PODXL expression have poorer survival.

The strength of this study is a large patient cohort with gastric cancer and a long follow-up with reliable clinical follow-up time and survival data. By the TMA technique, only small areas of the tumor are evaluated compared to whole-tissue sections. In colorectal cancer, Larsson et al. [22] found that use of whole sections allowed identification of a larger number of tumors as PODXL-positive than did TMA analysis. Because PODXL expression is heterogeneous, use of the TMA technique may lead to underestimation of positive cases. Scoring of both monoclonal-antibody and polyclonal-antibody expression was for some specimens difficult; cases with only a few cancer cells and those in which the value of the cancer cells was unidentifiable were thus excluded from analysis. This, in part, explains the high number of excluded cases. On the other hand, the TMA technique allows analysis of large patient cohorts with a homogeneous staining method.

In conclusion, PODXL immunostaining serves as an independent marker of poor prognosis in gastric cancer. This is, to our knowledge, the first report on the prognostic value of PODXL expression in gastric cancer. Future studies should confirm this association and resolve the mechanisms by which PODXL affects the development and behavior of gastric cancer.

## References

[1] Jemal A, Bray F, Center MM, Ferlay J, Ward E, Forman D (2011) Global cancer statistics. CA: A Cancer Journal for Clinicians 61: 69–90. 10.3322/caac.20107 21296855

[2] Dicken BJ, Bigam DL, Cass C, Mackey JR, Joy AA, Hamilton SM (2004) Gastric adenocarcinoma: review and considerations for future directions. Ann Surg 241: 27–39 10.1097/01.sla.0000149300.28588.23 PMC1356843 15621988

[3] Kerjaschki D, Noronha-Blob L, Sacktor B, Farquhar MG (1984) Microdomains of distinctive glycoprotein composition in the kidney proximal tubule brush border. J Cell Biol 98: 1505–1513. 6371023 10.1083/jcb.98.4.1505 PMC2113241

[4] Doyonnas R, Nielsen JS, Chelliah S, Drew E, Hara T, Miyajima A, et al (2005) Podocalyxin is a CD34-related marker of murine hematopoietic stem cells and embryonic erythroid cells. Blood 105: 4170–4178. 10.1182/blood-2004-10-4077 15701716

[5] Horvat R, Hovorka A, Dekan G, Poczewski H, Kerjaschki D (n.d.) Endothelial Cell Membranes Contain Podocalyxin—the Major Sialoprotein of Visceral Glomerular Epithelial Cells. The Journal of Cell Biology 102: 484–491. 3511072 10.1083/jcb.102.2.484 PMC2114082

[6] Somasiri A (2004) Overexpression of the Anti-Adhesin Podocalyxin Is an Independent Predictor of Breast Cancer Progression. Cancer Research 64: 5068–5073. 10.1158/0008-5472.CAN-04-0240 15289306

[7] Michael Schopperle W, Kershaw DB, DeWolf WC (2003) Human embryonal carcinoma tumor antigen, Gp200/GCTM-2, is podocalyxin. Biochemical and Biophysical Research Communications 300: 285–290. 10.1016/S0006-291X(02)02844-9 12504081

[8] Casey G (2006) Podocalyxin variants and risk of prostate cancer and tumor aggressiveness. Human Molecular Genetics 15: 735–741. 10.1093/hmg/ddi487 16434482

[9] Cipollone JA, Graves ML, Köbel M, Kalloger SE, Poon T, Gilks CB, et al (2012) The anti-adhesive mucin podocalyxin may help initiate the transperitoneal metastasis of high grade serous ovarian carcinoma. Clin Exp Metastasis 29: 239–252. 10.1007/s10585-011-9446-0 22262060

[10] Larsson AA, Johansson MEM, Wangefjord SS, Gaber AA, Nodin BB, Kucharzewska PP, et al (2011) Overexpression of podocalyxin-like protein is an independent factor of poor prognosis in colorectal cancer. Br J Cancer 105: 666–672. 10.1038/bjc.2011.295 21829192 PMC3188928

[11] Larsson A, Fridberg M, Gaber A, Nodin B, Levéen P, Jönsson G, et al (2011) Validation of podocalyxin-like protein as a biomarker of poor prognosis in colorectal cancer. BMC Cancer 12: 282–282. 10.1186/1471-2407-12-282 PMC3492217 22769594

[12] Kaprio T, Fermér C, Hagström J, Mustonen H, Böckelman C, Nilsson O, et al (2014) Podocalyxin is a marker of poor prognosis in colorectal cancer. BMC Cancer 14: 493 10.1186/1471-2407-14-493 25004863 PMC4226963

[13] Boman K, Larsson AH, Segersten U, Kuteeva E, Johannesson H, Nodin B, et al (2013) Membranous expression of podocalyxin-likeprotein is an independent factor of poorprognosis in urothelial bladder cancer. Br J Cancer 108: 2321–2328. 10.1038/bjc.2013.215 23652315 PMC3681027

[14] Saukkonen K, Hagström J, Mustonen H, Juuti A, Nordling S, Fermér C, et al (2015) Podocalyxin Is a Marker of Poor Prognosis in Pancreatic Ductal Adenocarcinoma. PLoS ONE 10: e0129012 10.1371/journal.pone.0129012 26053486 PMC4459962

[15] Heby M, Elebro J, Nodin B, Jirström K, Eberhard J (2015) Prognostic and predictive significance of podocalyxin-like protein expression in pancreatic and periampullary adenocarcinoma. BMC Clin Pathol 15: 10 10.1186/s12907-015-0009-1 26028992 PMC4449563

[16] Kaprio T, Hagström J, Fermér C, Mustonen H, Böckelman C, Nilsson O, et al (2014) A comparative study of two PODXL antibodies in 840 colorectal cancer patients. BMC Cancer 14: 494 10.1186/1471-2407-14-494 25004935 PMC4107962

[17] Uhlén M, Björling E, Agaton C, Szigyarto CA-K, Amini B, Andersen E, et al (2005) A human protein atlas for normal and cancer tissues based on antibody proteomics. Mol Cell Proteomics 4: 1920–1932. 10.1074/mcp.M500279-MCP200 16127175

[18] Pontén F, Jirström K, Uhlen M (2008) The Human Protein Atlas—a tool for pathology. J Pathol 216: 387–393. 10.1002/path.2440 18853439

[19] Wiksten J-P, Lundin J, Nordling S, Kokkola A, Haglund C (2008) Comparison of the prognostic value of a panel of tissue tumor markers and established clinicopathological factors in patients with gastric cancer. Anticancer Res 28: 2279–2287. 18751407

[20] Mrena J, Wiksten J-P, Kokkola A, Nordling S, Ristimäki A, Haglund C (2009) COX-2 is associated with proliferation and apoptosis markers and serves as an independent prognostic factor in gastric cancer. Tumor Biol 31: 1–7. 10.1007/s13277-009-0001-4 20237896

[21] Archie V, Kauh J, Jones DV, Cruz V, Karpeh MS, Thomas CR (2006) Gastric cancer: standards for the 21st century. Crit Rev Oncol Hematol 57: 123–131. 10.1016/j.critrevonc.2005.09.004 16412659

[22] Larsson AH, Nodin BR, Syk I, Palmquist I, Uhlén M, Eberhard J, et al (2013) Podocalyxin-like protein expression in primary colorectal cancer and synchronous lymph node metastases. Diagnostic Pathology 8: 1–1. 10.1186/1746-1596-8-109 23819542 PMC3751142

[23] Ponnusamy MP, Seshacharyulu P, Lakshmanan I, Vaz AP, Chugh S, Batra SK (2013) Emerging role of mucins in epithelial to mesenchymal transition. Curr Cancer Drug Targets 13: 945–956. 24168188 10.2174/15680096113136660100 PMC3924542

[24] Ponnusamy MP, Lakshmanan I, Jain M, Das S, Chakraborty S, Dey P, et al (2010) MUC4 mucin-induced epithelial to mesenchymal transition: a novel mechanism for metastasis of human ovarian cancer cells. Oncogene 29: 5741–5754. 10.1038/onc.2010.309 20697346 PMC3005772

